# Are *Bifidobacterium* Species Key Players in the Progression of Type 1 Diabetes? A Systematic Review

**DOI:** 10.1002/edm2.70120

**Published:** 2025-10-13

**Authors:** Vanina Vergoz, Donna Jeong, Emma E. Hamilton‐Williams

**Affiliations:** ^1^ School of Medicine University of Notre Dame Australia Sydney New South Wales Australia; ^2^ Frazer Institute The University of Queensland Brisbane Queensland Australia

**Keywords:** *Bifidobacterium*, microbiota, type 1 diabetes

## Abstract

**Background:**

Type 1 diabetes (T1D) frequently develops in childhood and is preceded by a non‐symptomatic period of autoimmunity. Alterations in the gut microbiome are implicated in T1D pathogenesis. *Bifidobacterium* is a significant focus due to its positive health impacts, association with breastfeeding and presence in probiotics and infant milk‐formulas. This systematic review aims to investigate *Bifidobacterium*'s association with T1D across disease stages.

**Methods:**

A comprehensive electronic search was conducted in MEDLINE, EMBASE and Web of Science, from 2011 to 2024. The search used a combination of medical subject headings and keywords related to *Bifidobacterium*. Studies included individuals at risk of T1D (pre‐stage, stage 1 or 2 asymptomatic T1D) and with stage 3 symptomatic T1D while excluding T2D, clinical trials and animal studies.

**Results:**

The search initially retrieved 1120 articles. Of these, 25 papers met the inclusion criteria, covering 4533 individuals (842 cases with or at‐risk of T1D and 3691 healthy controls). The studies highlighted variability in *Bifidobacterium* abundance in T1D, with higher abundance found more often in at‐risk asymptomatic individuals and lower abundance frequently found in those with established T1D.

**Conclusion:**

These findings do not support loss of *Bifidobacterium* as a key factor in the early development of T1D. Further studies are needed to explore *Bifidobacterium*'s role in T1D progression and management.

## Graphical abstract

*Bifidobacterium* are generally considered beneficial bacteria, and are found in probiotic formulations currently being trialled for type 1 diabetes (T1D) prevention. In this systematic review, we find that there is a reduction in *Bifidobacterium* species after T1D diagnosis and an increase or no change in *Bifidobacterium* abundance in those at risk of T1D. These findings do not support a role for *Bifidobacterium* in T1D prevention.

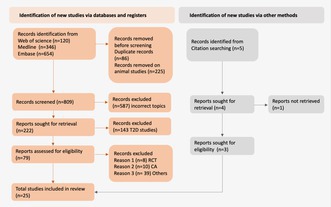

## 1 Introduction

Type 1 diabetes (T1D) is an autoimmune disease characterised by the progressive destruction of insulin‐producing pancreatic islet beta cells, leading to insulin dependence. Future risk of T1D can be identified by both the presence of genetic risk, mainly from the human leukocyte antigens (HLA) as well as the presence of islet‐specific autoantibodies. T1D is now recognised to occur in distinct stages, with the appearance of two or more islet‐specific autoantibodies (IAb) considered ‘stage 1’ T1D, followed by ‘stage 2’ T1D characterised by IAb and dysglycemia without symptoms and finally ‘stage 3’ symptomatic T1D [1].

The global number of T1D cases was estimated to be 8.4 million individuals in 2021 and is increasing [2]. While T1D exhibits a notable genetic component, the marked rise in its prevalence suggests environmental influences also play an important role in disease progression [3]. These environmental factors may include infant diet, mode of birth, viral infections, gut microbiota and breastfeeding, although evidence for each of these varies [3]. In support of a role for the gut microbiota in T1D progression, the Environmental Determinants of Diabetes in the Young (TEDDY) Study found that a reduction in bacteria that ferment fibre to produce short‐chain fatty acids in children that progressed to islet autoimmunity or T1D [4]. TEDDY also found that infants given probiotic bacteria in the first month of life had a decreased risk of progression to islet autoimmunity [5]. Thus, increasing abundance of certain beneficial probiotic bacteria may reduce T1D risk in young children.

Various *Bifidobacterium* species stand out as promising probiotics due to their ability to positively impact the health of their host, both directly and indirectly. *Bifidobacterium* species are important in infants for utilisation of human milk oligosaccharides [4]. Beneficial effects of *Bifidobacterium* have been reported to include protection against inflammation, hypertension and diabetes [6]. *Bifidobacterium* is thought to exert its influence on the host system by modulating the microbiome milieu and interacting with the host immune system, including early life imprinting of an immune‐regulatory environment [7, 8]. It is unknown whether increasing *Bifidobacterium* abundance would be beneficial in T1D and at which stage of disease, early as a preventative measure or later after clinical onset to aid in disease management.

Numerous clinical trials are investigating the therapeutic potential of probiotics, particularly *Bifidobacterium* species, including for the prevention of islet autoimmunity or aiding T1D management. A recent trial in type 2 diabetes showed that *Bifidobacterium* supplementation improved fasting glucose and HbA1C levels [9]. The Global Platform for the Prevention of Autoimmune Diabetes—SINT1A Study is exploring whether daily *Bifidobacterium infantis* EVC001 supplementation in infants at genetic risk for T1D can decrease the incidence of islet autoimmunity in childhood [10]. However, evidence in the literature for a reduced abundance of *Bifidobacterium* genus members is variable, highlighting the need for a systematic review to clarify *Bifidobacterium*'s role in T1D at different disease stages.

This systematic review aims to answer the question: is the abundance of *Bifidobacterium* species associated with disease in either symptomatic T1D individuals (stage 3 T1D) or in at‐risk individuals with either islet autoimmunity and/or genetic risk (pre‐stage, stage 1 and stage 2 T1D)? Through a comprehensive analysis, we seek to provide clarity regarding the role of *Bifidobacterium* in different stages of T1D and its potential implications for disease prevention and management.

## 2 Methods

This systematic review adhered to the guidelines outlined by PRISMA (Preferred Reporting Items for Systematic Reviews and Meta‐Analyses), with the protocol registered at PROSPERO under registration number CRD42023440838.

### 2.1 Literature Search

MEDLINE, Embase and Web of Science were searched using keywords including but not limited to ‘Gut microbiome, Type 1 diabetes’, ‘Type 1 diabetes, microbiome’, ‘islet autoimmunity’, ‘*Bifidobacterium*’ and ‘16S sequencing’ (Table S1) spanning from 2011 to 25/01/2025. Reference lists of previous systematic reviews [11, 12] and other papers identified within extracted papers were also reviewed.

### 2.2 Screening Strategy

Search results were imported into EndNote 20. Two reviewers (VV and DJ) independently applied eligibility and exclusion criteria (defined below) and reviewed titles and abstracts. They assessed the title and abstract of each paper in the database independently and selected the papers that conformed to the selection criteria for full text evaluation. If there were discrepancies, both reviewers discussed the issue with a third reviewer (EHW). Subsequently, identified papers were then manually screened to confirm their relevance to the topic and eligibility according to the criteria below.

### 2.3 Eligibility Criteria

1. Reporting relative and absolute abundance data of any *Bifidobacterium* species.
2. Utilising any method for data collection, including but not limited to 16S ribosomal RNA‐targeted amplicon sequencing, whole‐genome (shotgun) metagenomic sequencing or qPCR.
3. Written in English.
4. Encompassing populations of interest, that is, individuals with T1D or at risk of T1D, including those with persistent islet autoantibodies, with or without high‐risk HLA alleles compared to a control population.
5. Any age of participants, from birth to 65 years old. Studies utilising similar or the same cohorts and datasets, such as those extracted from the TEDDY or DIPP studies, were considered, with careful assessment to mitigate bias.

### 2.4 Exclusion Criteria Included

1. Animal studies.
2. Interventional studies (Randomised controlled trials (RCTs), non‐randomised trials).
3. Conference abstracts, reviews, systematic reviews, meta‐analyses, case reports, or case series.
4. Conference papers or editorials.

### 2.5 Data Extraction

Data extraction from eligible articles was performed by one reviewer (VV) and checked by an additional reviewer (EHW). Data extracted included first author, publication year, country, participant characteristics (sample size, mean age, sex ratio, disease stage) and sample sources. Disease stage was defined according to [1], with stage 0 as individuals with a family history or genetic risk without autoimmunity, stage 1 as the presence of two or more islet autoantibodies without symptoms, stage 2 as the presence of two or more islet autoantibodies and dysglycemia without symptoms, and stage 3 as symptomatic clinically diagnosed T1D. RNA/DNA extraction methods, analysis methods and major findings related to *Bifidobacterium* were tabulated. Discrepancies were resolved through consultation with a third reviewer to reduce reporting bias.

### 2.6 Quality Assessment

Two appraisers evaluated the included studies using the Joanna Briggs Institute (JBI) appraisal scale for observational, cohort and case–control studies [13]. Studies scoring 6 or higher were considered of sufficient quality for inclusion (Table S2).

## 3 Results

### 3.1 Study Selection

**FIGURE 1:**
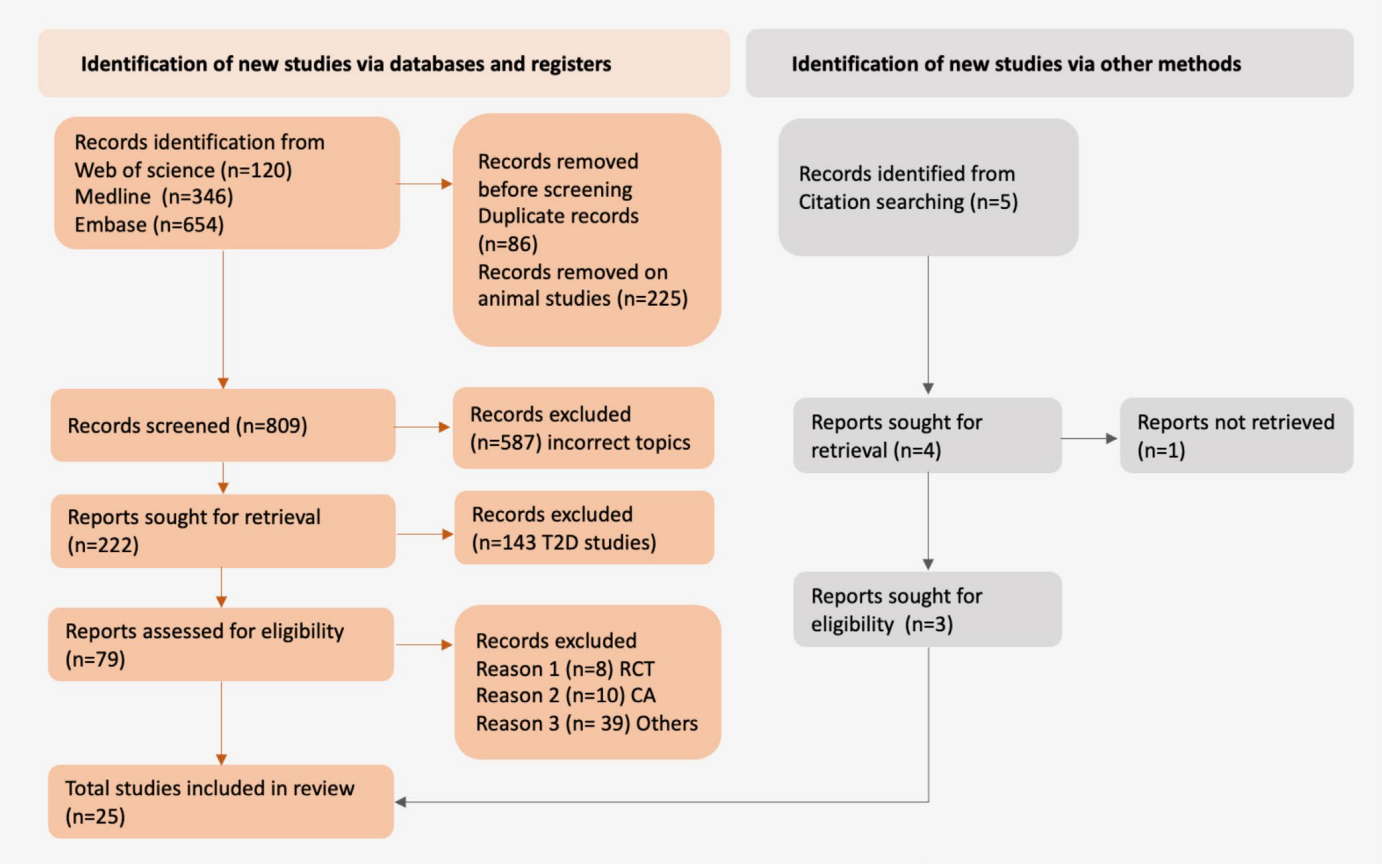
PRISMA flow chart of the studies included and excluded. CA, conference abstract; RCT, randomised control trial; T2D, Type 2 diabetes.

Following database searching, 1120 articles were retrieved: 654 from EMBASE, 346 from MEDLINE and 120 from Web of Science (Figure 1). After removing 225 animal studies and 86 duplicates, 809 study abstracts were screened, excluding 587 irrelevant papers. Of the remaining 222, 143 were excluded for focusing on type 2 diabetes, leaving 79 for detailed assessment. After evaluating these, 57 were excluded due to incorrect study type or low JBI scale scoring. Three additional papers were identified from references, resulting in 25 papers meeting the inclusion criteria for this systematic review.

### 3.2 Study Characteristics

The 25 selected articles included a total of 4533 individuals: 842 T1D cases (including pre‐T1D and stage 1–3 T1D) and 3691 healthy controls (HCs). Table 1 summarises the general characteristics of these studies conducted across 19 countries: India, Turkey, Tunisia, Finland, Estonia, Iran, Azerbaijan, Jordan, Nigeria, Sudan, Brazil, Spain, Italy, Poland, Netherlands, Russia, UK, USA and China. Most studies analysed faecal samples to investigate gut microbiota changes, with one study analysing oral samples and one analysing gut mucosa brushings. DNA amplification methods included quantitative PCR, metagenomic whole genome sequencing and amplicon sequencing of various 16S rRNA variable gene regions: V1–V2 (1 study), V2–V3 (2 studies), V3–V4 (5 studies) and V4 (1 study). Ten studies used the Illumina MiSeq platform, while two used the 454 platform. Data analysis commonly utilised QIIME and databases like Greengenes, RDP, MetaPhlAn2 and rrnDB. One study used culture‐based approaches.

**TABLE 1:** Overview of studies included.

Author, Year, Country	Study type	Size (*N* = case/control) T1D staging	Mean age	Methods
Biassoni 2020 Italy	Case–control study	*n* = 31/25 Stage 3	Average = 10.3 ± 4.1 years of age	16S rRNA gene sequencing (V2, V4, V8, V3, V6‐7, and V9 regions) Greengenes and MicroSEQ ID
Brown 2011 Finland	Case–control study	*n* = 4/4 Stage 1	Average = 150 days of life	Metagenomic whole genome sequencing Illumina Genome Analyser IIx
Chukhlovin 2023 Russia	Case–control study	*N* = 41/183 Stage 3	T1D mean age 18.2 and HC mean age 26.5	16S rRNA gene sequencing V3‐V4 region, Ilumina MiSeq platform/SILVA v138 database
Cinek 2018 Azerbaijan Jordan, Nigeria and Sudan	Case–control study	*n* = 73/103 Stage 3	T1D 11.7 years, interquartile range 7.8–13.7 and HC 11.3 years, interquartile range 8.1–13.7	16S rRNA gene sequencing V4 region, Ilumina MiSeq platform/SILVA database
Cinek^a^ 2016 Finland	Cohort study	*n* = 18/18 Pre‐stage ➔ Stage 1	3 up to 36 months of age (median 17.4 months)	16S rRNA gene sequencing V4 region, Ilumina MiSeq platform/SILVA database
Davis Richardson^a^ 2014 Finland, Estonia	Cohort study	*n* = 29/47 Pre‐stage ➔ Stage 1	Longitudinal: 4–6 months until 2.2 years of age	16S rRNA gene sequencing V3‐V4 regions Illumina HiSeq and MiSeq GreenGenes/QIIME database
De Groot 2017 Netherland	Case–control study	*n* = 53/50 Stage 3	Age 18–65 years old	16S rRNA gene sequencing V1‐V2 region Illumina MiSeq V2 platform QIIME database
Demerci 2022 Turkey	Case–control study	*n* = 40/40 Stage 3	Mean age 31 years old	qPCR
Ejtahed 2019 Iran	Case–control study	*n* = 21/40 Stage 3	Mean age 35.4 T1D and 38 for control	qPCR
Fassatoui 2019 Tunisia	Case–control study	*n* = 10/11 Stage 3	Age: 20–67 years old	qPCR
Kostic 2015 Finland, Estonia	Cohort study	*n* = 11/12 Pre‐stage to stage 3	From birth‐ 3 years old	16S rRNA gene sequencing V4 region and metagenomic whole genome sequencing Illumina MiSeq V2 platform GreenGenes QIIME database and MetaPhlAn 1.7.7
Krawczyk 2023 Poland	Case–control study	*n* = 33/33 Stage 3	Mean age 39.36 T1D and 41.21 for control	qPCR
Lakshmanan 2021 USA	Analytical cross‐sectional study	*n* = 24/5 Stage 3	Mean age 9.4 for case and 8.8 for control	16S rRNA gene sequencing V3‐V4 region Illumina MiSeq V2 platform Greengenes/QIIME Database
Leiva‐Gea 2018 Spain	Case–control study	*n* = 15/13 Stage 3	Mean age 12.56 for case and 12.25 for control	16S rRNA gene sequencing V2‐V3region/ 454 platform/ Greengenes/QIIME database
Liu 2021 China	Case–control study	*n* = 51/47 Stage 3	T1D mean age 10.38 ± 3.59/HC mean age 9.58 ± 4.35	16S rRNA gene sequencing V3‐V4 regions Illumina MiSeq platform QIIME
Lo Conte 2023 Italy	Observational cross‐sectional study	*n* = 17/16 Stage 3	T1D mean age = 34.5 ± 3 HC mean age = 43.3 ± 3.5	16S amplicon sequencing V3–V4 region Illumina Miseq platform Kraken2/NCBI Genome
Maffeis 2016 Italy	Case–control study	*n* = 10/10 Pre‐stage/Stage 1	Age 6–16 years old	PCR‐DGGE^c^ Partial 16SRNA gene sequence
Murri 2013 UK	Case–control study	*n* = 16/16 Stage 3	T1D mean age 7.16 ± 0.72 years, and HC mean aged 7.48 ± 0.87 year	PCR‐DGGE Partial V2‐V3 region 16SRNA gene sequence rrnDB database
Salamon 2018 Poland	Case–control study	*n* = 22/23 Stage 3	T1D mean age 36 year/HC mean age 37 year	16S rRNA gene sequencing V3‐V4 regions Illumina MiSeq platform Greengenes/QIIME database
Soyucen 2013 Turkey	Case–control study	*n* = 35/35 Stage 3	T1D mean age 10.73 ± 4.16 years and mean age for HC 9.96 ± 4.09 years	Colony/Bacteria cultured using both selective, quantitative methods and non‐ selective methods
Stewart^b^ 2018 USA and Europe	Cohort study	*n* = 196/632 The TEDDY study Pre‐stage to stage 3	3–46 months of age	16S rRNA gene sequencing, V4 region, Illumina MiSeq platform, SILVA database Metagenomic whole genome sequencing, Illumina HiSeq platform, MetaPhlAn2 database
Talukdar 2021 India	Analytical cross‐sectional study	*n* = 8/9 Stage 3	18–60 years of age	16S rRNA gene sequencing V3‐V4 regions Ilumina MiSeq platform RDP database
Traversi 2020 Italy	Case–control study	*n* = 40/56 Stage 3	Age 5–10 years old	PCR‐DGGE Partial V3‐V4 region 16S rRNA gene sequence qPCR
Vatanen^b^ 2018 USA and Europe	Cohort study	*n* = 114/418 The TEDDY study Pre‐stage to stage 3	3–46 months of age	Metagenomic whole genome sequencing Illumina HiSeq platform, MetaPhlAn2 database
Van Heck 2022 Netherland	Case–control study	*n* = 240/2937 Stage 3	Type 1 diabetes average age of 52 ± 16 years and HC 51.3 ± 164.7 years	Metagenomic whole genome sequencing Ilumina HiSeq 2000 platform MetaPhlAn2 database

^a^ Data extracted from a subset of participants from the DIPP study.

^b^ Data extracted from a subset of participants from the TEDDY study.

^c^ PCR‐DGGE: Polymerase chain reaction/denaturing gradient gel electrophoresis.

JBI quality scores for the eligible studies are shown in Table S2, with all scoring above 6, indicating satisfactory quality. The majority of studies (17 out of 25) scored 8 or above, indicating they were high‐quality studies.

### 3.3 Bifidobacterium in Individuals in Pre‐Stage and Stage 1 T1D

We first considered studies with samples collected from participants at increased risk of future progression to stage 3 T1D (Table 2). Data from the largest pre‐T1D/stage 1 study (TEDDY) showed no significant association of *Bifidobacterium* with islet autoimmunity (IA) using 16S rRNA amplicon sequencing [14]. Further analysis of a similar set of samples also from TEDDY using whole genome metagenomic sequencing identified variations in *Bifidobacterium dentium* abundance, which were higher in the control cohort compared to cases with islet autoimmunity [4]. In contrast, *Bifidobacterium pseudocatenulatum* was found at higher abundance in children with islet autoimmunity compared to their control counterparts [4]. Three other studies showed increased *Bifidobacterium* in individuals at high risk for T1D. Maffeis et al. found *Bifidobacterium longum* *subsp. longum* in 60% of prediabetic children, but none in controls [15]. Brown et al. noted higher *Bifidobacterium* abundance and other lactate‐producing bacteria in young children with two islet autoantibodies [16]. Davis‐Richardson et al. reported a modest increase (less than 10%) in *Bifidobacterium* in genetically at‐risk individuals progressing toward T1D [17]. Interestingly, Cinek et al. using a similar dataset (DIPP) with 16S rRNA gene sequencing, identified two operational taxonomy units (OTUs) inversely associated with children who later developed islet autoimmunity. Lower abundance of *Bifidobacterium bifidum* (adj *p* = 5.5 × 10^−6^) and *Bifidobacterium pseudocatenulatum* (adj *p* = 0.071) were associated with islet autoimmunity [18]. A longitudinal study by Kostic et al. tracked children from birth to 3 years old and noted a decrease in overall gut microbiota diversity before T1D onset, but no change in *Bifidobacterium* abundance [19]. Kostic et al. also observed increased Bifidobacterium during breastfeeding compared to post‐breastfeeding periods. In summary, four of eight studies found increases in *Bifidobacterium* species in at‐risk children, two studies found a decrease and two studies found no significant change (Table 2).

**TABLE 2:** Trends in abundance of *Bifidobacterium* in individuals in pre‐stage and stage 1 T1D.

Studies^a^	Year	Bifidobacterium in pre‐stage and stage 1 T1D compared to Control
Maffeis	2016	↑ *Bifidobacterium longum*
Brown^b^	2011	↑ Abundance at the phylum level of *Actinobacteria* + *Bifidobacterium* more abundant
Davis Richardson^b^	2014	↑ Relative abundance genus of *Bifidobacterium* (modest)
Vatanen^c^	2018	↓ *Bifidobacterium dentium* ↑ *Bifidobacterium pseudocatenulatum*
Cinek^b^	2016	↓ *Bifidobacterium pseudocatenulatum* and ↓ *Bifidobacterium bifidum*
Stewart^c^	2018	NS
Kostic	2015	NS

^a^ All studies were in paediatric populations.

^b^ Data extracted from a subset of participants from the DIPP study.

^c^ Data extracted from a subset of participants from the TEDDY study.

### 3.4 *Bifidobacterium* in Individuals With Stage 3 T1D

We next considered studies that sampled individuals after clinical T1D diagnosis (Table 3). Biassoni et al. found that newly diagnosed individuals had a higher relative abundance of *Bifidobacterium bifidum* compared to healthy controls [20]. Biassoni et al. also noted a positive correlation between age at onset and *Bifidobacterium* abundance and a negative correlation with anti‐insulin autoantibodies. Additionally, they found a positive correlation between Body Mass Index Standard Deviation Score (BMI SDS) and the abundance of *Bifidobacterium adolescentis* and *Bifidobacterium bifidum* . This suggests BMI is a relevant confounder, as it is a known risk factor for T1D.

**TABLE 3:** Trends in abundance of *Bifidobacterium* for individuals in stage 3 symptomatic T1D.

Study	Year	Bifidobacterium in T1D Compared to Control	Population
Biassoni	2020	↑ *Bifidobacterium bifidum*	Paediatric
Chukhlovin	2023	↓ *Bifidobacterium*	Adult
Demerci	2022	↓ *Bifidobacterium longum* gene expression NS *Bifidobacterium adolescentis*	Adult
Ejtahed	2019	↓ *Bifidobacterium*	Adult
Lakshmanan	2021	↓ Relative abundance of *Bifidobacterium adolescentis* and ↓ *Bifidobacterium longum* in T1D with HBP	Paediatric
Leiva‐gea	2018	↓ Relative abundance of *Bifidobacterium*	Paediatric
Lo Conte	2023	↓ Relative abundance of *Bifidobacterium dentium*	Adult
Murri	2013	↓ *Bifidobacterium*	Paediatric
Krawczyk	2023	↓ *Bifidobacterium*	Adult
Soyucen	2013	↓ *Bifidobacterium* colonisation	Paediatric
Van Heck	2022	↓ *Bifidobacterium longum*	Adult
Cinek	2018	NS	Paediatric
Traversi	2020	↓ *Bifidobacterium*	Paediatric
De Groot	2017	NS	Adult
Salamon	2018	NS	Adult
Fassatoui	2019	NS	Adult
Liu	2021	NS	Paediatric
Talukdar	2021	NS	Adult

Abbreviation: HBP, high blood pressure.

Several studies in stage 3 T1D consistently reported decreased Bifidobacterium. Chukhlovin et al. found a lower abundance of the *Actinomycetota* phyla and the *Bifidobacter* genus in individuals with T1D compared to the control group [21]. Additionally, they noted a negative correlation between *Bifidobacterium* and age, suggesting altered abundance may be more relevant in younger individuals. Demerci et al. found *Bifidobacterium longum* abundance was significantly reduced in adults with T1D compared to controls, while no significant difference was found with *Bifidobacterium adolescentis* [22]. Additionally, they noted a weak but significant positive correlation between *B. adolescentis* and *B. longum* and the gene expression of short‐chain fatty acid receptors GPR41 and GPR43 in T1D individuals' blood. Moreover, the T1D group exhibited significantly higher GPR43 expression compared to controls. Using 16S rRNA gene‐targeted primers specific for *Bifidobacterium*, Ejtahed et al. found decreased faecal *Bifidobacterium* load in adults with T1D compared to healthy controls (*p* = 0.04) [23]. Lakshmanan et al. reported lower relative abundance of *B. longum* and *B. adolescentis* in hypertensive children with T1D compared to their normotensive counterparts and non‐T1D controls [24]. Normotensive individuals with T1D, on the other hand, showed an increased abundance of *Bifidobacterium* genera compared to healthy controls [24].

Leiva‐Gea et al. found significantly decreased *Bifidobacterium* abundance in children with T1D compared to healthy controls, with a positive correlation between *Bifidobacterium* depletion and low serum IL‐10 and IL‐13 levels [25]. A recent study by Lo Conte et al. revealed that changes in the mucus layer of the small intestine observed in individuals with T1D were associated with a decreased relative abundance of *Bifidobacterium dentium* compared to healthy controls [26].

Another study by Krawczyk et al. found using quantitative PCR that the number of *Bifidobacterium* was significantly lower in individuals with T1D compared to their healthy counterparts [27]. Additionally, they identified a negative correlation between *Bifidobacterium* abundance and both glucose and HbA1c levels. Similarly, Murri et al. observed reduced faecal *Bifidobacterium* abundance in children with T1D compared to healthy controls, and this reduction correlated negatively with plasma glucose levels in T1D children [28]. Soyucen et al. demonstrated a significant decrease in faecal *Bifidobacterium* in children with recent‐onset T1D compared to healthy controls using selective culturing [29]. In a comprehensive metagenomic analysis, van Heck et al. found *B. longum* depletion in adults with long‐standing T1D [30].

In contrast to these findings, several other studies reported non‐significant changes in the abundance of *Bifidobacterium*. For instance, the study by Cinek et al. found no significant results, despite observing an overall decreasing trend in the relative abundance of *Bifidobacterium* in individuals with T1D from Azerbaijan and Sudan [31]. Furthermore, a large variability was noticed in the relative abundance of the 10 most abundant genera obtained from individuals from African and Asian countries [31]. Traversi et al. reported a lack of *Bifidobacterium* in stool samples from children with T1D (0%) compared to healthy controls (28.6%) using DGGE band sequencing [32]. However, 16S gene sequencing revealed that the presence of *Bifidobacterium* spp. had a protective effect against T1D onset (OR 0.20; 95% CI 0.10–0.38, *p* = 0.0001). In contrast, de Groot et al. found no significant difference in *Bifidobacterium* abundance between adults with T1D (60%) and controls (43%) [33]. Similarly, Fassatoui et al. observed no significant difference in *Bifidobacterium. longum* spp. abundance between a small group of adults with and without T1D [34].

In the study by Liu et al. children with established T1D showed no discernible difference in *Bifidobacterium* abundance compared to controls [35]. Similarly, Salamon et al. found no significant difference between the long‐standing T1D group and the control group. However, they noted a positive correlation between *Bifidobacterium* and high‐density lipoprotein cholesterol in T1D individuals [36]. Finally, Talukdar et al. observed no significant difference in *B. longum* abundance in adults with T1D [37]. In summary, 11 of 18 studies in stage 3 T1D reported a decrease in *Bifidobacterium* abundance, 6 studies found no change and 1 study reported an increase (Table 3). No specific pattern was observed in relation to whether the stage 3 studies were in adults versus children, suggesting the commonly observed reduction in *Bifidobacterium* abundance occurs in both adults and children.

## 4 Discussion

This review underscores *Bifidobacterium* variability in T1D, with frequently higher abundance in at‐risk individuals but lower abundance after diagnosis. The studies varied in demographics and T1D stages, posing challenges in interpretation. Four studies showed increased *Bifidobacterium* in early‐stage T1D, two found a decrease. Eleven reported lower abundance in stage 3, while one found an increase. Eight studies showed non‐significant results, mainly in stage 3. Despite these differences, *Bifidobacterium* abundance fluctuated across T1D stages and ages, complicating distinctions between these factors.

The decrease in the abundance of *Bifidobacterium* in individuals with T1D after clinical onset appears to be the most consistent trend observed across the different studies. Individuals with long‐standing T1D likely have additional T1D comorbidities such as micro‐ and/or macrovascular complications linked to hyperglycemia that can significantly influence the composition of the gut microbiome [30]. Even a slight reduction in *Bifidobacterium* can correspond with factors contributing to disease severity, including increased GPR43 receptor expression [22], elevated blood pressure [24], reduced levels of anti‐inflammatory cytokines like IL 10 and IL 13 [25], and elevated plasma glucose [28] or HbA1c [27]. It remains unclear whether altered *Bifidobacterium* may directly impact disease progression or merely show associations.

*Bifidobacteria* operate within a complex microbial community, interacting with various bacterial species. For example, *B. adolescentis* facilitates cross‐feeding among bacteria via lactate and acetate production, contributing to butyrate formation by other gut microbes [38, 39]. Short‐chain fatty acids (SCFAs) like butyrate, acetate and propionate are crucial for maintaining epithelial barrier integrity and regulating host immune responses [40]. While *Bifidobacterium's* negative association with T1D post‐diagnosis is noteworthy, increasing its abundance may not necessarily provide a benefit. Eight studies found no significant difference in *Bifidobacterium* abundance between individuals with T1D and controls [19, 33, 34, 35, 36, 37], suggesting a more complex interplay within the microbial community.

Davis‐Richardson et al. noted an upregulation of *Bacteroides* in infants before seroconversion [17]. A recent metaproteomic study associated this upregulation of *Bacteroides* with downregulation of *Bifidobacterium*‐related KEGG functions in young individuals with T1D compared to controls [41, 42]. Supporting this, Biassoni et al. (2020) found elevated *Bacteroides stercoris* , *Bacteroides fragilis* and *Bacteroides intestinalis* alongside increased *Bifidobacterium* abundance in newly diagnosed T1D patients. This indicates that even with higher *Bifidobacterium* abundance in young individuals with T1D, their function may be compromised by a dysregulated environment, such as increased abundance of *Bacteroides*. In addition, *Bifidobacterium* produces acetate [43], a SCFA known to affect the expression of SCFA receptors GPR41 and GPR43, renowned for their anti‐inflammatory effects [44]. However, the effect of acetate on these receptors varies based on intracellular pathways and cell types expressing them [44]. *Bifidobacterium*'s role in T1D dynamics may depend on various factors, including but not limited to the profile of the microbiome environment [41, 42], and the types of SCFA receptors expressed in β cell islets [45].

Studies reporting increased abundance of *Bifidobacterium* were mainly in children transitioning from pre‐stage to stage 1 T1D [15, 16, 17, 46]. Although the increase in *Bifidobacterium* abundance was often modest, one relatively small study found a correlation between three microorganisms ( *Dialister invisus* , *Gemella sanguinis* and *B. longum* ) and increased intestinal permeability in individuals at risk of T1D [15]. This contradicts the protective effect typically associated with *Bifidobacterium* but aligns with the concept of gut leakiness in T1D. Individual *Bifidobacterium* species have unique functions, with numbers fluctuating with age, most notably species associated with breastfeeding. Thus, the difference in patterns observed between stages of T1D may be confounded by age.

Whether or not increasing the abundance of *Bifidobacterium* species can prevent islet autoimmunity or T1D onset or improve glucose control in individuals with symptomatic T1D is still an outstanding question. A small number of systematic reviews have investigated randomised controlled trials of prebiotics, probiotics and synbiotics on glucose control in individuals with symptomatic T1D. Findings from these reviews have been mixed, with both improved [47, 48], worsened [49] and variable effects [50] on glycemic indices reported. No systematic reviews have investigated probiotic use in children at risk of T1D, likely due to the lack of relevant prevention trials. However, the degree to which any of these effects were due to changes in *Bifidobacterium* abundance is unclear, and further research is needed.

This systematic review has several limitations worth noting. Firstly, the included studies employed diverse methods to assess the gut microbiome, such as 16S rRNA sequencing and qPCR, which can introduce variability based on targeted variable regions and primers used. Metagenomic analysis is generally less biased but was not uniformly utilized. Furthermore, many studies focused only on the genus level of Bifidobacterium without delving into species‐level analysis, typically necessitating metagenomics for accuracy. The variability in methods used to quantify Bifidobacterium and subsequent variability in the measures reported meant that it was not possible to perform a meta‐analysis across the studies. Moreover, factors like diet, age and time of day can significantly influence gut microbiome composition, which were not consistently accounted for. Finally, some studies had relatively small sample sizes, potentially affecting the robustness of their findings.

## 5 Conclusion

This systematic review highlights the significant variability in *Bifidobacterium* abundance associated with T1D. Individuals at risk of T1D often exhibit higher abundances of *Bifidobacterium* prior to diagnosis, followed by lower abundance after clinical onset. While *Bifidobacterium* may contribute to disease dynamics, it represents only one component in a complex biological system. These findings do not support the notion that reduced *Bifidobacterium* abundance is a key early driver in the onset of T1D. Further research is needed to clarify *Bifidobacterium's* role in T1D progression and management.

## Author Contributions

**Vanina Vergoz:** conceptualization (equal), data curation (lead), formal analysis (lead), investigation (equal), writing – original draft (equal). **Donna Jeong:** formal analysis (equal), investigation (equal). **Emma E. Hamilton‐Williams:** conceptualization (equal), supervision (equal), writing – review and editing (equal).

## Conflicts of Interest

The authors declare no conflicts of interest.

## Supporting information


**Data S1:** edm270120‐sup‐0001‐Tables.xlsx.

## Data Availability

The authors have nothing to report.
